# Cyberbullying: a storm in a teacup?

**DOI:** 10.1007/s00787-017-0954-6

**Published:** 2017-02-10

**Authors:** Dieter Wolke, Kirsty Lee, Alexa Guy

**Affiliations:** 1 Department of Psychology, University of Warwick, Coventry, CV4 7AL UK; 2 Warwick Medical School, University of Warwick, Coventry, CV4 7AL UK

**Keywords:** Cyberbullying, Bullying, Victimization, Self-esteem, Aggressive behavior, Interpersonal relationships

## Abstract

Cyberbullying has been portrayed as a rising ‘epidemic’ amongst children and adolescents. But does it create many new victims beyond those already bullied with traditional means (physical, relational)? Our aim was to determine whether cyberbullying creates uniquely new victims, and whether it has similar impact upon psychological and behavioral outcomes for adolescents, beyond those experienced by traditional victims. This study assessed 2745 pupils, aged 11–16, from UK secondary schools. Pupils completed an electronic survey that measured bullying involvement, self-esteem and behavioral problems. Twenty-nine percent reported being bullied but only 1% of adolescents were pure cyber-victims (i.e., not also bullied traditionally). Compared to direct or relational victims, cyber-victimization had similar negative effects on behavior (*z* = −0.41) and self-esteem (*z* = −0.22) compared to those not involved in bullying. However, those bullied by multiple means (poly-victims) had the most difficulties with behavior (z = −0.94) and lowest self-esteem (*z* = −0.78). Cyberbullying creates few new victims, but is mainly a new tool to harm victims already bullied by traditional means. Cyberbullying extends the reach of bullying beyond the school gate. Intervention strategies against cyberbullying may need to include approaches against traditional bullying and its root causes to be successful.

**Electronic supplementary material:**

The online version of this article (doi:10.1007/s00787-017-0954-6) contains supplementary material, which is available to authorized users.

## Implications and contribution

Cyberbullying creates very few new victims. The majority of cyber-victims are bullied traditionally, i.e., directly or relationally in their peer group. Adolescents that are bullied experience more behavior and self-esteem problems and those bullied by various means (poly-victims) are the most severely affected. Intervention efforts should need to include a focus on traditional bullying.

“Cyber-bullying: Horror in the home” [1], and “It’s time to stop the cyberbullying epidemic” [2], are some of the headlines claiming that social media have created a new demon: cyberbullying. Indeed, within the past 10 years, the number of research articles published on this topic has risen exponentially, with some claiming cyberbullying to be a ‘new phenomenon’ created by the availability of electronic media which is an increasing problem for children and adolescents [3, 4].

However, others have criticized the hype surrounding cyberbullying, believing this to be a largely overrated phenomenon [5]. There are at least two issues that need to be addressed to be certain that we are facing a new epidemic. Firstly, does cyberbullying create new victims, or is it another tool in the armory to bully those who are already victims of traditional bullying at school? Secondly, does cyberbullying in adolescence have unique effects on psychological and psychosocial outcomes, above what is experienced by victims of traditional bullying?

Cyberbullying is broadly defined as bullying that is carried out via electronic means such as text messages, emails, online chatrooms or social networking sites [6]. The reported prevalence of cyberbullying amongst adolescents varies considerably, ranging from as low as 5–10% [7] to 50% [4], or as high as 72% [8]. There may be real variations due to differential use of electronic media across regions or schools, or because of measurement issues, according to a recent review [9]. But how many cyber-victims are also bullied by traditional means? Juvonen and Gross [8] found that 85% of cyber-victims were also traditional victims. Olweus [5] reported on two studies showing co-occurrence of traditional and cyberbullying of 88–93% and similar rates were recently reported by others [10, 11]. This suggests that 9 out of 10 adolescents who report experience of cyber-victimization are also bullied by traditional forms of bullying [6, 12]. Such considerable overlap rates may further account, at least in part, for the considerable variation in prevalence reported for cyberbullying and strongly suggests that cyberbullying is an extension of traditional bullying, i.e., it is a new weapon for bullies to use against targets they also bully at school.

Cyber-victimization has been associated with depression, anxiety, stress, self-esteem and behavioral problems in adolescence and beyond [13, 14], with some claiming the outcomes for cyber-victims may be even worse than for traditional victims [3, 15]. This may be partly because most cyber-victims are also victims of traditional bullying, so it is important for researchers to control for traditional victimization. There is some evidence that cyberbullying may have unique negative effects on self-esteem, and increase depression and anxiety symptoms [16]. But are the effects of cyberbullying worse, equivalent or less severe than traditional bullying, or is it that those who are victimized via multiple means, i.e., poly-victims, suffer the worst consequences [17]? A recent study suggests that adolescents who reported they had been both cyber and traditionally victimized had the highest emotional difficulties, peer and conduct problem scores [18]. Thus, being victimized in several ways may increase the risk of adverse psychological outcomes [19].

The aims of the current study were to assess the prevalence of cyberbullying occurring independently of traditional bullying, and the unique association of being a cyber victim on self-esteem and behavior difficulties in a large sample of adolescents from UK secondary schools.

## Methods

### Design and sample

A power analysis, conducted by averaging prevalence rates of traditional and cyberbullying [5, 7, 20], and using normative data on the Strengths and Difficulties Questionnaire (SDQ) [21], indicated that a minimum of 1983 participants were required for the study to detect a small effect size for cyber-victimization (*d* = 0.3) at.80 power. Attrition in school-based studies of bullying occurs at a rate of approximately 30%, thus we aimed to ask a minimum of 2833 pupils to participate.

**Fig. 1:**
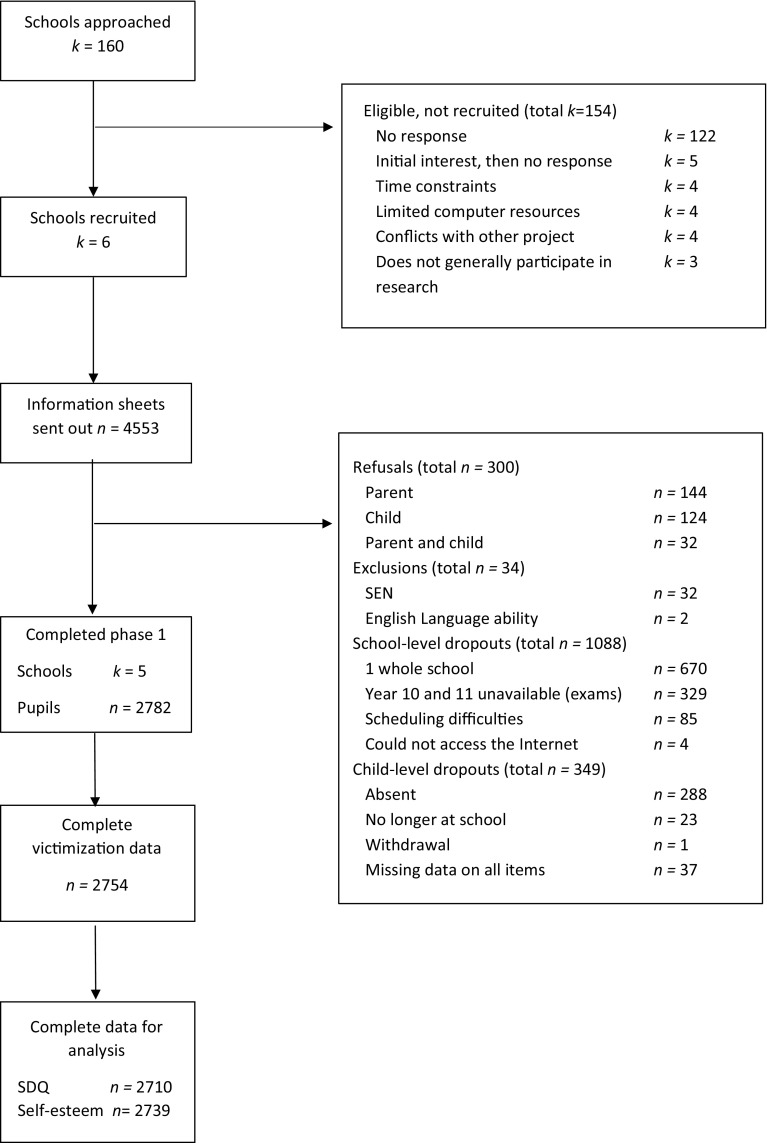
STROBE flow diagram of recruitment and selection of schools and participants

Adolescents aged 11–16 years (*M* = 13.5, SD = 1.35) attending mixed and single sex secondary schools in the Midlands, UK were assessed. The majority were White British (82.5%) and female (56.9%). Six schools originally agreed to participate but one subsequently dropped out. In the five remaining schools 3883 pupils were enrolled. We invited all pupils to participate, meaning the recruitment and participation rate was higher than planned. 2782 (71.6%) consented to participate and 2754 had complete data on the victimization items, as shown in the STROBE diagram [22] in Fig. 1. The main reasons for dropouts were parent and child refusals or school absence during data collection.

## Measures

### Peer bullying

Pupils completed the Bullying and Friendship Interview schedule [23], which has been used in numerous studies to assess bullying and victimization [24, 25]. The scale uses behavioral descriptions only; at no point was the term bullied or bullying used. There were five items on *direct* victimization (DV), e.g., “been hit/beaten up”, “called bad/nasty names”, and four items on *relational* victimization (RV), e.g., “had nasty lies/rumors spread about you”, “been made to do things you did not want to” assessing traditional victimization (i.e., those that experience direct and/or relational victimization in school). Four items asked about *cyber*-victimization (CV): “had rumors spread about you online”, “had embarrassing pictures posted online without permission”, “had private emails, messages or photos forwarded to someone else or where others can see it”, and “got threatening or aggressive emails, instant messages, text messages or tweets”. Pupils were asked how often each behavior had occurred within the last 6 months (never, occasionally [1–3 times], often [more than 4 times], or frequently [at least once a week]). Pupils who responded “never” or “occasionally” were categorized as non-victims. Pupils who responded “often” or “frequently” to any item (*n* = 807; 29.3%) were categorized as victims and seven distinct victim types could be distinguished: [1] pure direct victims (pure DV); [2] pure relational victims (pure RV); [3] pure cyber-victims (pure CV); [4] direct and relational victims (DV & RV); [5] direct and cyber-victims (DV & CV); [6] relational and cyber-victims (RV & CV); or [7] direct, relational, and cyber-victims (DV, RV, & CV). Grouping in this way allowed a comparison in outcomes across each possible victim type. By definition, multiple types of victimization will mean a higher frequency of victimization (see Supplementary File 1).

### Self-esteem and behavior difficulties

Self-esteem was assessed with the Rosenberg [26] Self-Esteem Scale, a 10-item measure answered on a 4-point scale (0 = *disagree a lot*, 3 = *agree a lot*). Responses to each item were summed (total scores ranging from 0 to 30), with higher scores indicating higher self-esteem. Behavioral and emotional difficulties were assessed with the Strengths and Difficulties Questionnaire (SDQ) [27], a widely used measure in 11–17 year olds to screen for psychiatric problems with good reliability and validity [28]. The 25-item measure is answered on a 3-point scale (0 = *not true*, 2 = *certainly true*), and has five distinct subscales: hyperactivity, emotional symptoms, peer problems, conduct problems, and prosocial behavior. Scores from all subscales, excluding the prosocial behavior subscale, were summed to generate a total difficulties score (ranging from 0 to 40), where higher scores indicate more difficulties. Two items were removed before computation of the total score to avoid overlap of constructs (i.e., “I fight a lot. I can make other people do what I want” and “Other children or young people pick on or bully me”), as they relate to bullying and victimization.

## Control variables

### Child-level factors (level 1)

Pupils self-reported their sex, age, and ethnicity. Ethnicity was dichotomized into White British vs. Minority, as there were too few participants in each ethnicity category to allow meaningful comparisons (White Other = 3.8%, mixed ethnicity = 4.1%, Asian = 6.1%, Black = 3.3%). Schools provided data on school year [7–11] and attendance rate (percentage).

### Family-level factors (level 1)

Pupils self-reported their parent’s highest level of education (high school, college, university: i.e., =<11, 12–13, or >13 years of education, respectively). Schools provided data on pupil premium status, i.e., additional funding that schools receive to raise attainment in disadvantaged pupils, including pupils who are currently or historically (within the past 6 years) eligible for free school meals. Pupil premium is therefore a family-level indicator of deprivation or special financial assistance.

### School-level factor (level 2)

All pupils were nested within a school, so “School” was included as a level 2 control variable, accounting for the hierarchical nature of the data.

## Procedure

Head teachers were approached in writing with full details of the study. Written information sheets were sent to pupils and parents in sealed envelopes. Parents returned an opt-out form if they did not want their child to participate. Data were collected from pupils in class-sized [20–35] groups during one lesson (50–60 min). At the start of each session pupils were given standardized instructions, assured about confidentiality, and gave their informed, written consent. The electronic questionnaire was accessible through individual passwords. Demographic questions were asked first and the remaining measures were counterbalanced. Once complete, children were redirected to an online game for the remainder of the session. For data quality purposes adolescents only completed the questionnaire whilst the researchers were present. Data collection took place between October 2014 and July 2015.

The study was approved by the University of Warwick’s ethics committee.

## Analysis

Dropout analysis was conducted to assess differences between participants and dropouts (i.e., refusals and child-level dropouts; Fig. 1). A dummy variable was created (0 = *participant*, 1 = *dropout*) and bivariate analyses (Chi-square comparisons, *t* tests) were computed on sex, school year, pupil premium status and attendance. A missing data analysis was conducted to evaluate whether missing data was related to peer victimization, self-esteem and behavior difficulties, or control variables. Missing data were dummy coded (0 = *responded*, 1 = *missing*) and bivariate analyses were computed. The dropout and missing data analyses informed the inclusion of relevant control variables in the modeling. For the first research question, victim type frequencies were calculated; for the second research question, a series of multilevel models were run using Maximum Likelihood estimation. Models were built up sequentially: model 1 was the crude association of the predictor (victim type) with the outcome (self-esteem; SDQ); model 2 adjusted outcomes after inclusion of the Level 1 control variables and the Level 2 variable (School), accounting for the nested structure of the data. School was included as a random factor, because schools were regarded as a sample of a larger population of schools and to test for level 2 effects. In all analyses the non-victim group was used as the reference category, except for planned a priori analyses (with a Sidak correction) of pure CV to the other victim types on SDQ and self-esteem scores. All analyses were computed using SPSS version 22.

## Results

### Dropouts, missing data, and descriptive statistics

Dropouts (*n* = 649) were older, had lower school attendance rates, and were more likely to have pupil premium status (Table 1). Pupils with missing data on all items of the predictor or any outcome variable were excluded from the analysis (*n* = 37; 1.3%). Missing data (*n* = 238; 8.6%) was not associated with victim type, but was associated with ethnicity, age, parent education, pupil premium status, and attendance.

**Table 1:** Descriptive data and associations with child- and family-level control variables for pupil-level dropouts and refusals; total participants and for each victim type

	Dropout vs. participants			Victim types (*n* = 2754)								
Variable	Dropouts	Participants		Non-victims	Pure DV	Pure RV	Pure CV	DV & RV	DV & CV	RV & CV	DV, RV, & CV	
*n*	649	2782		1947	222	159	31	205	24	25	141	
	%		*p*	%								*p*
Child-level factors												
Sex			0.087									.037
Female	53.0	56.8		57.4	49.1	54.7	58.1	54.1	54.2	80.0	63.8	
Male	47.0	43.2		42.6	50.9	45.3	41.9	45.9	45.8	20.0	36.2	
School year			<0.001									.002
7	19.9	25.3		25.3	27.0	23.9	25.8	27.8	16.7	0	23.4	
8	23.3	24.3		25.7	20.3	18.2	28.8	23.4	20.8	20.0	18.4	
9	19.9	21.3		19.8	27.5	25.8	3.2	27.3	25.0	20.0	27.0	
10	24.5	19.1		18.7	18.5	20.8	29.0	15.1	25.0	40.0	21.3	
11	12.5	10.0		10.5	6.8	11.3	16.1	6.3	12.5	20.0	9.9	
Mean age [95% CIs]	–	13.51 [13.46, 13.56]		13.48 [13.43, 13.55]	13.46 [13.29,13.63]	13.57 [13.36, 13.79]	13.83 [13.27, 14.38]	13.34 [13.17, 13.52]	13.86 [13.27, 14.44]	14.42 [13.97, 14.87]	13.71 [13.50, 13.93]	.002
Ethnicity												>.250
White British	–	82.5		82.0	83.4	86.0	93.5	80.5	87.5	92.0	85.1	
Minority	–	17.5		18.0	16.6	14.0	6.5	19.5	12.5	8.0	14.9	
Mean % attendance [95% CIs]	91.36 [92.47, 93.64]	95.05 [95.39, 95.75]	<0.001	95.78 [95.57, 95.98]	95.73 [95.15, 96.32]	95.18 [94.42, 95.95]	94.72 [93.21, 96.22]	95.06 [94.29, 95.82]	95.18 [93.63, 96.73]	93.19 [90.49, 95.88]	94.33 [93.39, 95.28]	.001
Family-level factors												
Parent education	–											.024
<=11 years	–	12.3		11.5	14.4	13.2	6.5	13.2	12.5	16.0	19.9	
12–13 years	–	55.5		54.5	58.6	52.8	61.3	63.9	62.5	44.0	53.9	
> 13 years	–	32.2		34.0	27.0	34.0	32.3	22.9	25.0	40.0	26.2	
Pupil premium^a^			<0.001									<.001
No	71.1	78.8		81.8	73.2	79.2	74.2	71.1	79.2	62.5	70.4	
Yes	28.9	21.2		18.2	26.8	20.8	25.8	28.9	20.8	37.5	29.6	

All numbers are percentages, unless otherwise stated

*DV* direct victims, *RV* relational victims, *CV* cyber-victims. Values in brackets are 95% confidence intervals. Some data were unavailable for pupils who dropped out

^a^Pupil premium is an indicator of deprivation or special assistance used within schools

Victim type was associated with all of the child-level variables, except ethnicity and attendance (Table 1). Girls experienced all types of victimization more often than boys, except for pure direct victimization (pure DV): girls were more likely than boys to be relational and cyber-victims (RV & CV). The RV & CV group were older, had lower attendance rates, and were more likely to have pupil premium status.

### What is the prevalence of pure cyber-victimization?

**Fig. 2:**
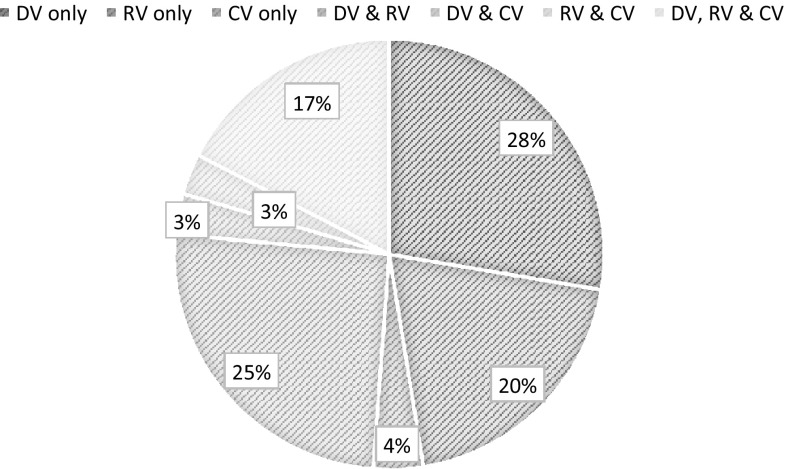
Pie chart of the frequencies (in percentages) of each victim type (includes victims only; *n* = 807)

Of all pupils, 29.3% were victims of bullying (Table 1). Pure DV was the most prevalent victim type, followed by DV & RV (Fig. 2). Traditional victimization (pure DV, pure RV, DV & RV) accounted for 73% of all victimization. Pure cyber-victimization was rare (4% of all those victimized); cyber-victimization occurred with traditional victimization 85.2% of the time.

### Do pure cyber-victims have more behavior and self-esteem difficulties than traditional victims?

All victims had lower self-esteem and more behavior difficulties compared to non-victims (Table 2: crude models), even after controlling for child and family-level factors (Table 2: adjusted models; see table footnotes for significant control variables). Pure CV had similar associations as pure DV & pure RV, meaning any type of pure victimization was related to lower self-esteem and more behavior difficulties. However, victims of multiple types of victimization had the lowest self-esteem and most behavior difficulties, particularly those who experienced both forms of traditional victimization, i.e., DV & RV, and those that experienced all three types of victimization (DV, RV, & CV).

**Table 2:** Crude and adjusted multilevel regression models to predict self-esteem and SDQ total behavior difficulties from victim type

	Self-esteem				Behavior difficulties (SDQ)				
	Crude model		Adjusted model^a^		Crude model		Adjusted model^b^		
	B [95% CI]	*p*	B [95% CI]	*p*	B [95% CI]	*p*	B [95% CI]		*p*
Intercept	19.40 [19.17, 19.63]	<.001	11.33 [6.57, 16.09]	<.001	11.14 [10.87, 11.40]	<0.001	24.73 [19.26, 30.21]		<.001
Victim type									
Pure DV	−2.62 [−3.35, −1.90]	<.001	−2.79 [−3.51, −2.07]	<.001	3.97 [3.14, 4.79]	<.001	4.00 [3.17, 4.82]		<.001
Pure RV	−1.60 [−2.45, −0.76]	<.001	−1.56 [−2.40, −0.73]	.005	3.14 [2.17, 4.11]	<.001	2.95 [1.98, 3.93]		<.001
Pure CV	−2.69 [4.54, −0.84]	.004	−2.19 [3.95, −0.44]	.004	4.63 [2.50, 6.75]	<.001	4.13 [2.08, 6.18]		<0.001
DV & RV	−4.64 [−5.39, -3.89]	<.001	−4.58 [−5.31, −3.84]	<.001	6.28 [5.42, 7.13]	<.001	5.96 [5.11, 6.81]		<.001
DV & CV	−3.03 [−5.13, −0.92]	.004	−2.89 [−4.88, −0.87]	.014	4.95 [2.57, 7.32]	<.001	4.59 [2.30,6.88]		<.001
RV & CV	−4.48 [−6.54, −2.42]	<.001	−2.87 [−4.87, −0.87]	<.001	7.46 [5.14, 9.79]	<.001		5.95 [3.65, 8.24]	<.001
DV, RV, & CV	−6.10 [−6.99, −5.21]	<.001	−5.34 [−6.22, −4.47]	<.001	8.37 [7.36, 9.38]	<.001	7.54 [6.53, 8.55]		<.001

Non-victims were the reference category. Crude models include the predictor (victim type) on each outcome variable. Adjusted models controlled for level 1 child and family variables (sex, ethnicity, parent education, pupil premium status (an indicator of deprivation) and percentage attendance) and included school as a level 2 (nested), random factor

*DV* direct victims, *RV* relational victims, *CV* cyber-victims. Values in brackets are 95% confidence intervals

^a^All level 1 control variables were significant: higher self-esteem was predicted by sex (boys), age (younger), attendance (higher) (*p* < .001), ethnicity (minority) (*p* = .002), pupil premium (no) (*p* = .035) and parent education (12–13 years; college level) (*p* = .011). The level 2 control variable (school) was not significant (*p* = .236)

^b^Except for parent education (*p* = .073) all level 1 control variables were significant (*p* < .001): higher total difficulties were predicted by sex (female), age (older), ethnicity (White British), attendance (lower), and pupil premium status (yes). The level 2 control variable (school) was not significant (*p* > .250)

**Fig. 3:**
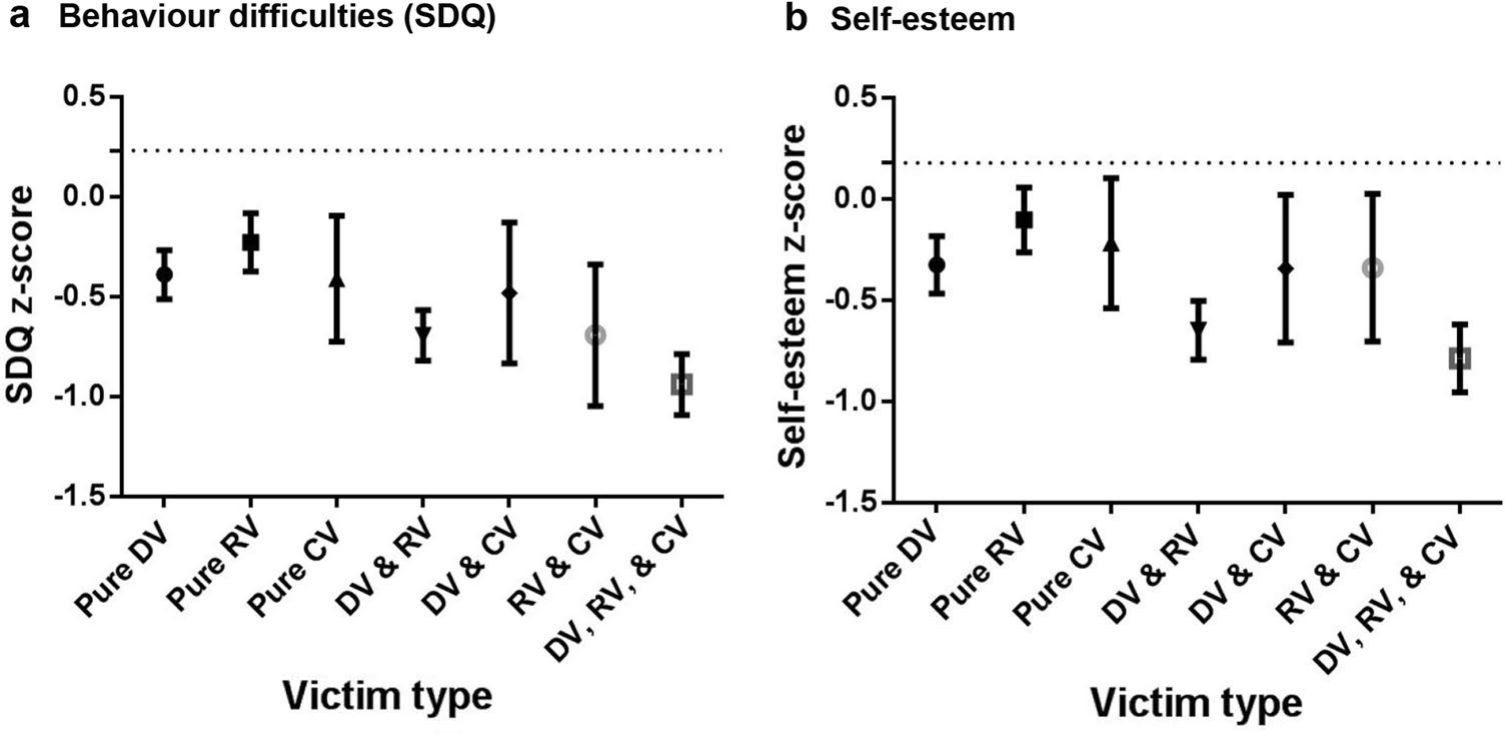
Transformed (z-scores of total population) crude SDQ total behavior difficulties and reversed self-esteem scores by victim type, with 95% confidence intervals

The a priori comparisons comparing pure CV to the other victim types revealed that pure CV had significantly higher self-esteem (*p* = .008) and fewer total difficulties on the SDQ (*p* = .034) than poly-victims (DV, RV, & CV), but their outcomes were not significantly different from the other victim types. Comparison between the victim groups, the effect sizes of the differences to non-victims and 95% confidence intervals are shown in Fig. 3.

## Discussion

The aim of this study was to investigate the unique prevalence and impact of cyberbullying in adolescence, in comparison to traditional bullying. In this sample of 11–16 year olds, pure cyber-victimization was very rare at around 1% of the total pupil population and 4% of victims of bullying. Cyber-victimization occurred mostly alongside traditional types of school bullying, such as direct and relational bullying. In terms of outcomes, pure cyber-victims had similar outcomes to pure direct victims and pure relational victims. Those who experienced poly-victimization by different means had the lowest self-esteem and most behavioral difficulties.

The finding of few pure cyber-victims found in this UK sample of adolescents is consistent with the low prevalence rates recently reported by other studies that assessed both traditional and cyber-victimization in the USA [6, 11]. Traditional or ‘in-person’ victimization was most prevalent, with almost all victimization being carried out by using direct or relational means. The majority of adolescents who reported experience of cyber-victimization were also victimized via these traditional means, supporting evidence that cyberbullying creates few new victims [10]. In this respect, these findings provide further evidence that cyberbullying is another tool in the toolbox for bullies. It should be seen as an extension of in-person bullying and not the unique or distinct phenomenon which has been portrayed [29, 30].

Regarding the impact upon psychological and psychosocial outcomes, pure cyber-victimization had similar effects as pure direct and pure relational victimization. Thus, any type of victimization is related to poorer psychological outcomes; namely, more behavioral and emotional difficulties and lower self-esteem. Furthermore, in accordance with other findings [17, 18], those who are victimized via multiple forms, in particular via multiple traditional forms (DV & RV, or DV, RV & CV), have especially low self-esteem and high behavioral difficulties.

Why do our and other recent research findings contradict the headlines of an epidemic of cyberbullying and its particular tragic consequences? Firstly, early research on cyberbullying [13] failed to assess traditional bullying, so effects on self-esteem or behavior were confounded by the most common types of peer victimization that adolescents experienced: direct and relational bullying. Secondly, the prevalence rates reported often over-shadow other important information regarding the participants and definitions used. For example, 94% of the adolescents assessed by Juvonen and Gross [8] had access to or use of the internet at home, and the 72% prevalence reported in this study was based upon experiencing one or more incidences of online ‘bullying’ in the past year. However, a single incidence of online harassment in one year should not be considered as bullying according to recognized consensus definitions that the aggressive acts have to be repeated [31].

It is important to understand that bullying occurs in peer relationships and is not an individual characteristics construct such as conduct disorder. Bullying is about exerting dominance and power to attain access to resources [32]. In adolescence, this includes dating and forming romantic relationships and those who are victimized have less romantic success than the bullies [33]. Indeed, bullying is one strategy to reduce intra-sexual competition, i.e., to defame and exclude competitors [34]. Understanding the evolutionary function of bullying requires that the bully is in the same environment and seen to be dominant to obtain access to the resources. Bullies also like to see the effects of bullying, i.e., the suffering of the victim and social isolation [35]. This is achieved by traditional means as shown here, and by also using new electronic means. Thus, cyberbullying on its own is very rare. It is not surprising that a recent review has shown that the risk factors for becoming a bully or victim of traditional and cyberbullying are very similar or identical [36].

Finally, our findings are consistent with previous reports that females engage more in relational bullying [37] and are more likely to be cyber-victims [7]. This may be explained by female adolescents spending more time on social media in contact with peers [38], meaning there is more opportunity, and that cyberbullying is similar in nature to female-dominated relational bullying, i.e., disrupting social relationships rather than confronting the victim directly. Moreover, those of lower socioeconomic status, indicated by pupil premium, were more likely to be victimized consistent with findings of a recent meta-analysis [39].

This study has a number of strengths. It involved a large sample of adolescents with experience of victimization and used reliable and valid measures to investigate bullying experiences, emotional and behavioral difficulties and self-esteem. Participants were provided with behavioral descriptions for acts of traditional and cyber-victimization, and a stringent criterion was used of including only those frequently or often victimized.

There are also limitations of the study. Firstly, this study focused on comparative frequency of cyber versus traditional victimization and is not representative of the UK as a whole. However, the prevalence and pattern of associations (such as with sex) are highly consistent with other UK-wide research previously reported [20]. Secondly, the nature of the association between victimization type and self-esteem and behavioral difficulties in this cross-sectional study is correlational and we therefore cannot infer any causation from the findings. However, there are now longitudinal and genetically sensitive studies [40] that have shown that being victimized by peers has adverse effects that are as detrimental as being abused by adults [41], get under the skin [42], and last a lifetime [43, 44]. To ascertain the effects of cyberbullying, in particular, future longitudinal research is needed [12].

To conclude, traditional types of school victimization remain the most frequent type of peer victimization amongst adolescents. Although pure cyber-victimization had similar psychological outcomes to pure direct and relational victims, poly-victims had the highest risk of poor psychological functioning. From a public health perspective, considering the low prevalence of pure cyber-victimization compared to traditional peer victimization, cyber-victimization has only a small unique impact on adolescent mental health; it is an overrated phenomenon. Cyberbullying is another means for traditional bullies to gain dominance and access to resources. Schools must acknowledge and address this issue, despite incidences often occurring outside of the school grounds. However, any bullying prevention and intervention still needs to be primarily directed at combatting traditional bullying while considering cyberbullying as an extension that reaches victims outside the school gate and 24/7.

## Electronic supplementary material

Below is the link to the electronic supplementary material.

Supplementary material 1 (DOCX 110 kb)

## Abbreviations

DV: Direct victims
RV: Relational victims
CV: Cyber-victims
CI: Confidence intervals
