# Supplementary material for: Cyberbullying: a storm in a teacup?
Source: Eur Child Adolesc Psychiatry. 2017 Feb 10;26(8):899–908. doi: 10.1007/s00787-017-0954-6 (PMC5532410; doi:10.1007/s00787-017-0954-6)
Supplement: Supplementary file 1 — Supplementary material 1 (DOCX 110 kb) [file 787_2017_954_MOESM1_ESM.docx]

**Supplementary file**

**Table 1** Mean total victimization score (with standard deviation) for each type of victimization type (total sample)

|  |  | Victim types | | | | | | | |  | |
| --- | --- | --- | --- | --- | --- | --- | --- | --- | --- | --- | --- |
|  |  | *Non-victims* | *Pure DV* | *Pure RV* | *Pure CV* | *DV & RV* | *DV & CV* | *RV & CV* | *DV, RV, & CV* | |  |
| Total victimization score (Mean ± SD) |  | 1.46 (1.83) | 5.77 (2.70) | 5.67 (2.61) | 6.12 (2.79) | 10.33 (3.82) | 10.05 (3.77) | 9.54 (3.66) | 16.71 (6.85) | |  |

*Note:* The total victimization score was computed by summing the responses to the 13 victimization items (range 0-39); pupils were asked how often they had been victimized directly (5 items), relationally (4 items) and through cyber means (4 items) within the last six months (never=0; occasionally [1-3 times]=1; often [more than 4 times]=2; or frequently [at least once a week])=3). Pupils who responded “often” or “frequently” to any item (*n*=807; 29.3%) were categorized as victims and seven distinct victim types could be distinguished: (1) pure direct victims (pure DV); (2) pure relational victims (pure RV); (3) pure cyber-victims (pure CV); (4) direct and relational victims (DV & RV); (5) direct and cyber-victims (DV & CV); (6) relational and cyber-victims (RV & CV); or (7) direct, relational, and cyber-victims (DV, RV, & CV). Grouping in this way allowed a comparison in outcomes across each possible victim type.


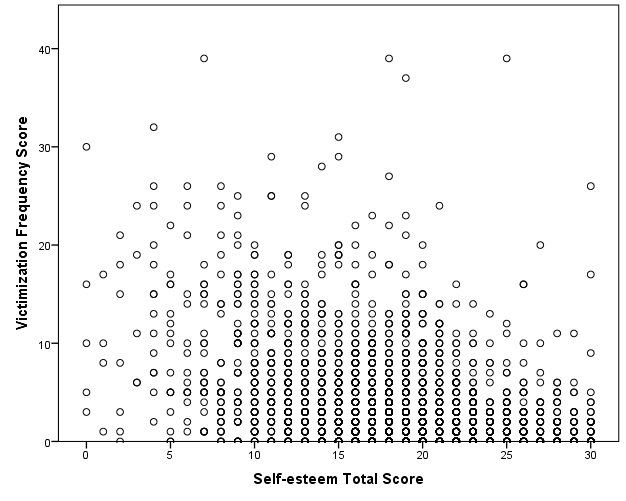


***(r=-.378, n=2706, p<.001)***

**Figure 1** Scatter diagram of the association between total victimization frequency and self-esteem total scores.


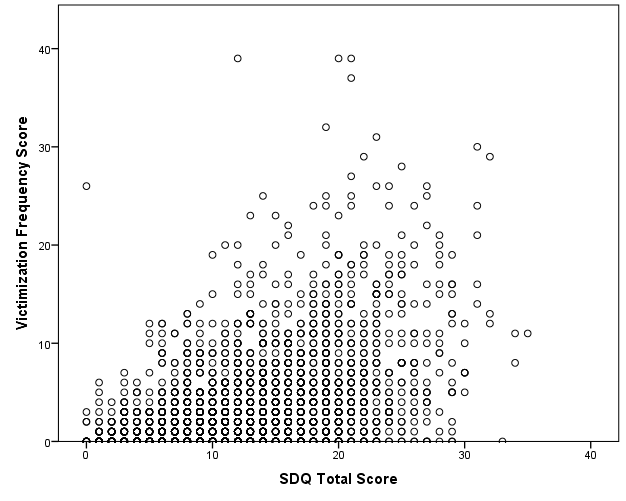


***(r=.460, n=2677, p<.001*)**

**Figure 2** Scatter diagram of the association between total victimization frequency and SDQ total scores.
